# Simultaneous Bioaugmentation and Biostimulation to Remediate Soil Contaminated by Ship Dismantling in Bangkalan District, Indonesia

**DOI:** 10.5696/2156-9614-9.24.191212

**Published:** 2019-11-27

**Authors:** Rizqi Nadhirawaty, Harmin Sulistiyaning Titah

**Affiliations:** Department of Environmental Engineering, Faculty of Civil, Environmental and Geo Engineering, Institut Teknologi Sepuluh Nopember (ITS), Surabaya, Indonesia

**Keywords:** bioremediation, consortium, metal, ship dismantling, soil, TPH

## Abstract

**Background.:**

High concentrations of total petroleum hydrocarbons (TPH), iron (Fe), and manganese (Mn) were identified in soil samples from two shipyards where vessel dismantling activities take place in Tanjungjati Village, Indonesia, and subjected to bioremediation.

**Objectives.:**

The aim of the present study was to determine whether the combination of surfactant solution, bioaugmentation (a consortium of Bacillus subtilis and Acinetobacter lwoffii), and biostimulation (nutrient amendment and aeration intermittent) would reduce TPH, Fe, and Mn levels from soil contaminated from ship dismantling activities.

**Methods.:**

Iron and Mn bioavailability were examined according to the Indonesian technical guidelines for soil chemical analysis with the help of atomic absorption spectrophotometry. The n-hexane solvent soil was extracted using the ultrasonic water bath method for TPH analysis.

**Results.:**

The highest removal results achieved were TPH (69.62%), Fe (87.10%), and Mn (29%) for Soil 1 samples and elimination of TPH (28.80%), Fe (65.10%), and Mn (57.38%) for Soil 2 samples using a combination of surfactant solution, bioaugmentation, and biostimulation (nutrient amendment and without aeration intermittent). Iron and Mn removal in the controls was higher than in the treated soils, which showed that Fe and Mn could decrease naturally in both contaminated soils.

**Conclusions.:**

The present study showed that bioremediation using a combination of surfactant solution, a consortium of Bacillus subtilis, and Acinetobacter lwoffii, as well as a nutrient amendment, has the potential to degrade hydrocarbons in contaminated soil. Furthermore, Bacillus subtilis and Acinetobacter lwoffii consortium used for bioaugmentation have the potential to enhance the degradation of hydrocarbons in soil.

**Competing Interests.:**

The authors declare no competing financial interests.

## Introduction

Tanjungjati Village in the Bangkalan district, Madura Island, Indonesia is known for its ongoing ship dismantling activities. Ship dismantling yards take old non-operational vessels and process them into products that may be sold back into the market chain. The main objective of ship dismantling activities is to obtain steel as a raw material for new ships. The dismantling activities in Tanjungjati Village consist of three stages: ship arrival and administration, pre-cutting (ship cleaning), and cutting. At the pre-cutting stage, the vessel is cleaned of flammable materials such as fuel, paper, rope, and plastic. Subsequently, decoating of the vessel is done by sandblasting to reduce the risk of fire in subsequent stages. The decoating process removes sand and metal particles from the air. Ship body cutting is conducted using the oxy-acetylene technique, which is considered to have lower risks than the use of oxy-liquified petroleum gas and manual techniques.^1^

Dismantling is carried out directly onshore, producing hazardous materials that can pollute the environment. These materials include polycyclic aromatic hydrocarbons, polyvinyl chloride, polychlorinated biphenyl, heavy metals, and tributyltin.^2^ Hydrocarbon compounds usually originate from tanks, pipes, machines, ballast water, waste fuel, oil, and grease produced during the cleaning stage.^3^ In addition to marine pollution, soil and sediment quality has also decreased due to hydrocarbons and metals.

Bioremediation is a soil remediation technique which uses microorganisms to degrade soil contaminants through biological activities. There are three general principles in bioremediation practice, including stimulation of the indigenous soil bacteria (biostimulation), such as nutrient amendment and supplying oxygen (aeration); exogenous bacteria additions (bioaugmentation); and intrinsic or natural attenuation with requirements, such as a high population of microorganisms, nutrient bioavailability, and environmental conditions, which lead to organism growth and efficient contaminant degradation.^4–6^ Studies show that biostimulation, bioaugmentation, or the combination of the two are effective in reducing pollutant concentrations in the soil, especially hydrocarbons.^7–9^

Previous research succeeded in isolating Bacillus subtilis from diesel-contaminated seawater on Kenjeran Beach, and Acinetobacter lwoffii from diesel-contaminated soil at a ship dismantling site in Tanjungjati, Bangkalan.^10^,^11^ B. subtilis is capable of degrading hydrocarbons and can survive in the presence of metals.^12–14^ In addition, A. lwoffii is a soil microorganism with the ability to decompose hydrocarbons.^15^,^16^ Currently, there are no studies on the use of these bacteria in bioaugmentation applications to soil contaminated with hydrocarbons and metals. The purpose of the present study was to determine the bioremediation potential to total petroleum hydrocarbons (TPH) and metals from ship dismantling-contaminated soil. The study uses a combination treatment of surfactant solution, bioaugmentation (a consortium of Bacillus subtilis and Acinetobacter lwoffii), and biostimulation (nutrient amendment and intermittent aeration).

## Methods

**Figure 1:**
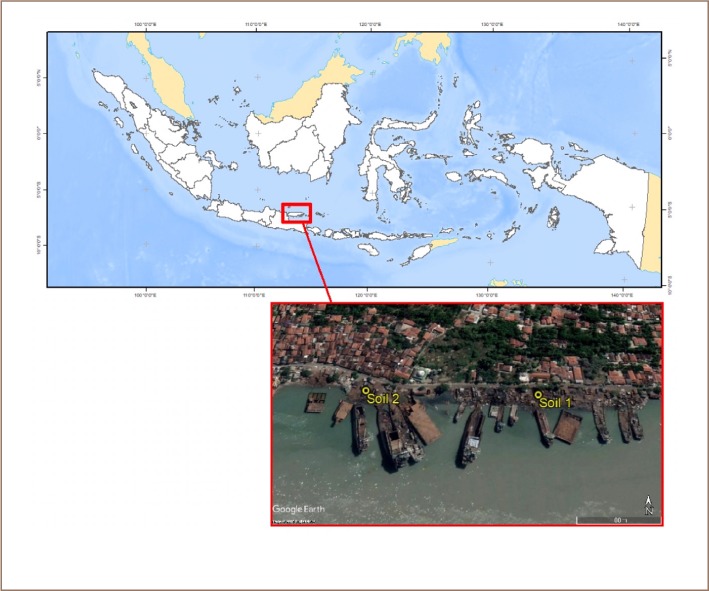
Map of Indonesia showing sampling sites (source: Google Earth, 2018)

Samples were collected from two ship dismantling yards in Tanjungjati Village; Soil 1 location (7°10′16.06″ S; 112°44′3.84″ E) and Soil 2 location (7°10′15.83″ S; 112°43′55.79″ E) *(Figure 1).* Twelve sampling points were used to obtain composite soil from both locations. At each sampling point, the samples were taken with a hand auger at a depth of 0–30 cm.

**Abbreviations**
*1A*: Soil 1 + intermittent aeration
*1AV*: Soil 1 + intermittent aeration + consortium + surfactant + nutrient
*1T*: Soil 1 − control
*1TV*: Soil 1 + consortium + surfactant + nutrient
*2A*: Soil 2 + intermittent aeration
*2AV*: Soil 2 + intermittent aeration + consortium + surfactant + nutrient
*2T*: Soil 2 − control
*2TV*: Soil 2 + consortium + surfactant + nutrient
*CO_2_*: Carbon dioxide
*TPH*: Total petroleum hydrocarbons

### Soil physical-chemical analysis

Soil parameters, such as pH, water content, organic carbon, total nitrogen, total phosphorus, phosphorus available, total potassium, texture, and metals bioavailability (iron (Fe), manganese (Mn), lead (Pb), cadmium (Cd) bioavailable) were analyzed according to the Indonesian technical guidelines for soil chemical analysis.^17^ Afterward, the samples were agitated with ethylenediaminetetraacetic acid 0.05 N solvent (pH adjust 7.00) for 1 hour and filtrated prior to atomic absorption spectrophotometry analysis (Agilent 240FS AA, Santa Clara, CA). The soil was n-hexane solvent extracted using the ultrasonic water bath method for TPH analysis.^18^ Ten (10) g of soil, anhydrous sodium sulfate, and 35 mL n-hexane were placed in a 100 mL bottle (Duran, Germany). Extractions were conducted for 60 minutes, at 50°C in an ultrasonic water bath (Krisbow, Indonesia). The extract was filtrated with glass wool and 25 mL n-hexane was added and made up to 60 mL final volume of supernatant, which was put into a flask and kept in a fume hood for 3–4 days.

### Bacteria preparation consortium

Bacillus subtilis and Acinetobacter lwoffii have been evaluated in the previous studies.^10^,^11^ The strains were inoculated in selective media Bacillus differentiation agar (Himedia, India) and Herellea agar (Himedia, India), and incubated for 24 hours in a 37°C incubator (Memmert, United Kingdom). Afterward, both the B. subtilis and A. lwoffii were inoculated in nutrient broth (Merck, Germany) and placed on a shaker with 150 rpm at room temperature (28 + 0.2°C) for 13 and 8 hours, respectively. Media selective for A. lwoffii and B. subtilis were used to determine both bacteria species with the pour plate technique during the bioremediation process.

### Bioremediation experiment

**Figure 2:**
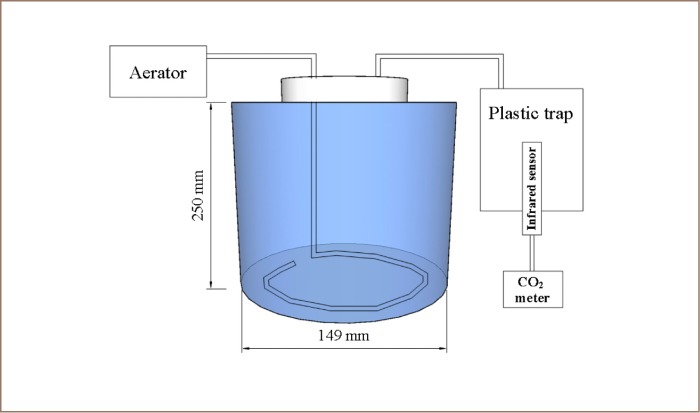
Reactor design for bioremediation experiment

The bioremediation design experiment was carried out in two replicates. It was conducted on a laboratory scale using a 4 L glass reactor with a diameter of 149 mm and height of 250 mm *(Figure 2).* Tween 80 solution (10 mg/L) was added first into both soil samples (10% vol/wt) and homogenized, then kept at room temperature (28 + 1°C) for 72 hours. Surfactant was added to increase the availability of contaminant, especially hydrocarbons. Each soil sample (250 g) was put into the glass reactor separately. Subsequently, nutrient (urea and superphosphate) were amended to 100:10:1 of carbon:nitrogen:phosphorus ratio. The consortium was inoculated into the soil by direct placement of 10% (vol/vol) nutrient broth (1:1 of A. lwoffii:B. subtilis), while intermittent aeration was periodically conducted once a day for 60 minutes with a flowrate of 1 L/min. Bioremediation was carried out after 42 days at room temperature (27.2 + 1°C). Soil moisture was maintained by 60% by adding distillate water twice a week. Carbon dioxide (CO_2_) measurements were taken using a portable meter (Lutron GC-2028, Taiwan), and the glass jars were closed for 16 hours with a plastic trap on caps. The mean CO_2_ concentrations were obtained after 150-second measurements with the value read after every 15 seconds, as shown on the instrument display.

Bioremediation experiments included: soil 1 + intermittent aeration + consortium + surfactant + nutrient (1AV); soil 1 + consortium + surfactant + nutrient (1TV); soil 2 + intermittent aeration + consortium + surfactant + nutrient (2AV); soil 2 + consortium + surfactant + nutrient (2TV); soil 1 + intermittent aeration (1A); soil 1 − control (1T); soil 2 + intermittent aeration (2A); and soil 2 − control (2T).

### Statistical analysis

The effects of bioremediation on the concentrations of TPH, Fe, and Mn were determined via one-way analysis of variance using the Statistical Package for the Social Sciences software (SPSS) version 21. Parameters of pH, CO_2_ concentration, and total colony number were analyzed using Spearman correlation.

## Results

Table 1 shows that both samples were composed of slightly alkaline, sandy loam soil with low nutrient content, except for phosphorus compounds, compared to data interpretation of the Indonesian technical guidelines for soil chemical analysis.^17^ Critical limit or level is defined as the maximum acceptable concentration of metals without long-term effects on the ecosystem. The critical limit of soil is also related to its impact on organisms and plants.^19^ Maize grown on soils with Fe and Mn approaching the critical limit had the highest accumulation of metals. However, maize's metal absorption ability was decreased when grown on soil which exceeded the critical limit. Lead and Cd in high concentrations are toxic to plants and inhibit soil urease activity and nitrification.^20^ The critical level of Fe, Mn, Pb, Cd in sandy soil is 3.4 mg/kg, 1.4 mg/kg, 64 mg/kg, and 5.5 mg/kg, respectively.^19^,^21^ Based on chemical analysis for Soil 1 and Soil 2 samples, the concentrations of Fe and Mn were above the critical levels. However, Cd and Pb concentrations were below significant levels.

**Table 1:** Soil Properties

**Soil properties**	**Soil 1**	**Soil 2**
Texture	Sandy loam	Sandy loam
pH-H_2_O	7.93	7.91
Water content (%)	6.43	4.35
Organic carbon (%)	1.88	2.82
Total nitrogen (%)	0.11	0.08
Phosphorus available (ppm)	155.45	16.14
Total phosphorus (mg P_2_O_5_/100g)	144.34	98.06
Total potassium (mg P_2_O_5_/100g)	3.75	1.53
Mg-exchangable (cmol/kg)	0.61	0.35
Fe-available (mg/kg)	703.1	226.3
Mn-available (mg/kg)	45.2	54.1
Pb-available (mg/kg)	8.8	11
Cd-available (mg/kg)	0.1	0.3
TPH (%)	7.29	9.51
Water holding capacity (%)	17	7

Abbreviation: H_2_O, water

Total petroleum hydrocarbon levels were 7.29% and 9.51% in Soil 1 and 2, respectively. The TPH levels in both soil samples were significantly above the maximum requirement (TPH 1%), according to Indonesian Environment Ministry Decree, No. 128 (2003).^22^ Soil 2 also contained more organic carbon and fewer nutrient compounds compared to Soil 1. In addition, the water holding capacity of Soil 1 was higher compared to Soil 2. This indicates that the hydrocarbon in the liquid phase filled most of the soil pores. Additionally, it indicates that Soil 2 lost moisture and had less oxygen between its pores compared to Soil 1. The pH value of both soils was still in the range to biologically degrade hydrocarbons in soil (pH 6–8).^23^

**Figure 3:**
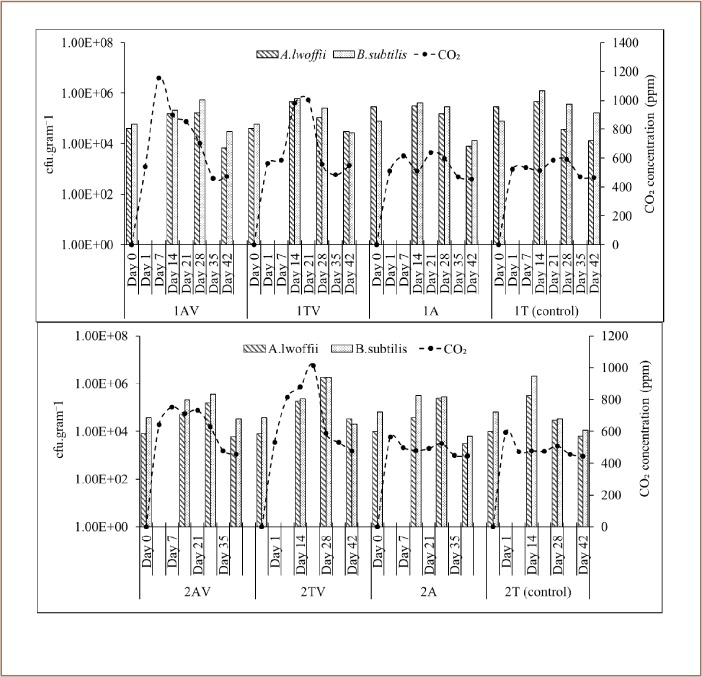
CO_2_ concentration, B. subtilis and A. lwoffii colony number

Figure 3 shows the highest CO_2_ concentrations in 1AV and 2AV had been achieved in the first 7 days of incubation, while the concentration of A. lwoffii and B. subtilis colonies in both reactors increased until the 28^th^ day. However, they started to decrease until the end of the bioremediation experiment, which was on day 42. In contrast, the highest CO_2_ concentrations in the 1TV and 2TV reactors was achieved on the 21^st^ day. The largest concentration of bacterial colonies occurred on the 14^th^ day and 28^th^ day in 1TV and 2TV reactors, respectively, followed by a decrease on the 42^nd^ day. After 21 days, the concentration of CO_2_ decreased on 1AV, 1TV, 2AV, and 2TV reactors, even though there was no significant difference between the 28^th^ day and the 42^nd^ day. In reactors that only received intermittent aeration treatment without bacterial consortium, the CO_2_ concentrations and the concentration of A. lwoffii and B. subtilis colonies did not change significantly from the beginning to the end of the bioremediation experiment, and the value was almost the same as in the control reactor (soil 1=1T and soil 2 = 2T).

In 1AV, 1TV, 2AV, and 2TV reactors, a surfactant solution was applied to the sample before the addition of a bacterial consortium. Figure 3 shows the concentration of bacterial colonies in the reactor without the addition of a consortium (1A, 2A), including controls (1T and 2T). The average value of bacterial colonies in the reactor without the addition of a consortium (1A, 1T, 2A, 2T) was not significantly different from the reactor with a bacterial consortium (1AV, 1TV, 2AV, 2TV). B. subtilis and A. lwoffii indigenous in Soil 1 decreased to 85.92% and 25.32%, respectively, after 72 hours of surfactant solution application. However, B. subtilis and A. lwoffii indigenous in Soil 2 decreased to 17.71% and 43.08%, respectively.

**Figure 4:**
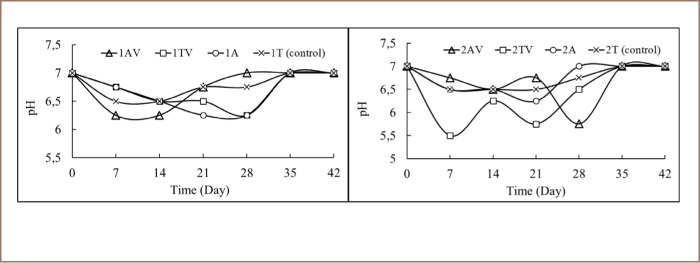
pH soil during bioremediation

The 1AV and 2AV reactors had acidic soil pH in the first 2 weeks, which rose close to 7 on the 21^st^ day *(Figure 4).* Furthermore, 1AV reactor had a neutral and stable pH until the 42^nd^ day. However, the soil pH of 2AV reactor decreased to 5.8 on the 28^th^ day, then became neutral on the 35^th^ to 42^nd^ day. The soil pH in 1TV and 1A reactors decreased until the 28^th^ day, and neutral conditions began to be evident from the 35^th^ day, a condition that also occurred in 2A reactor. The soil in 2TV reactor changed significantly on the 7^th^ day, then increased to 6.3 on the 14^th^ day before dropping to pH 5.8 on the 21^st^ day. During the 4^th^ week, the soil pH of 2TV reactor increased to neutral on the 35^th^ day. In the control reactor (1T, 2T), soil pH was never lower than 6.5, and between the 5^th^ week and the end of the bioremediation experiment, soil pH was 7.

**Figure 5:**
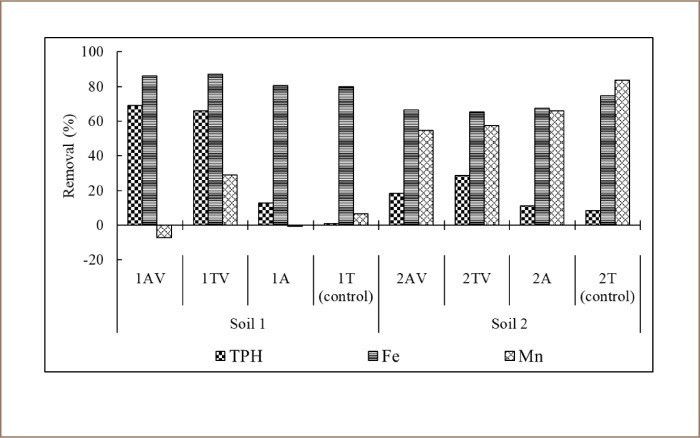
Contaminant removal after bioremediation

There was a correlation between CO_2_ concentration, pH, and A. lwoffii and B. subtilis colony number. There was a significant negative correlation between CO_2_ concentration and pH (r = −0.644; p < 0.05). This indicated that the increase of CO_2_ concentration was always followed by a decrease in pH value during bioremediation (*Figure 4*). A negative correlation was also evident between pH for both species of bacteria colony numbers. Total colony number of A. lwoffii to pH value was r = −0.288 (p > 0.05) and B. subtilis to pH value was r = −0.083 (p > 0.05). This indicated that the increase in colony number of both species followed a decrease in pH value. Figure 5 represented the removal of the contaminants after 42 days of bioremediation processes. For Soil 1 samples, 1AV had the highest removal of TPH (69.62%), while 1TV showed the highest elimination of Fe (87.10%) and Mn (29%). For Soil 2, 2TV had the highest removal of TPH (28.80%), while 2T (control) had the most significant elimination of Fe (74.51%) and Mn (83.71%).

The concentrations of contaminants in both Soil 1 and Soil 2 samples, and the combination of surfactant solution, bioaugmentation (consortium B. subtilis and A. lwoffii), and biostimulation (nutrient amendment and intermittent aeration) significantly contribute to bioremediation performance for reducing hydrocarbon, Fe, and Mn concentrations (p < 0.05). Surfactant solution, consortium B. subtilis and A. lwoffii, as well as a nutrient amendment, significantly affected hydrocarbon removal only (p < 0.05). The reactors with intermittent aeration (1AV, 1A, 2AV, 2A) showed slightly better hydrocarbon removal, no difference in Fe elimination, but worse Mn removal compared to reactors with no aeration (1TV, 1T, 2TV, 2T).

## Discussion

The present study sought to determine whether the combination of surfactant solution, bioaugmentation, and biostimulation has the potential to decrease the concentration of TPH, Fe, and Mn in soil contaminated by ship dismantling activities in Tanjungjati Village, Bangkalan district, Indonesia.

In both Soil 1 and Soil 2 samples, the levels of TPH, Fe, and Mn exceeded recommended maximum levels. The lower nutrient content and higher organic carbon of Soil 2 was due to its higher hydrocarbon compound content. The presence of hydrocarbon compounds in soil could increase organic matter, carbon, nitrogen, and also decrease the level of phosphorus, magnesium, calcium, sodium, and potassium.^24^,^25^ Moreover, the presence of hydrocarbon compounds in soil pores decreased water holding capacity and was characterized by lower water holding capacity in Soil 2 compared to Soil 1.

Although there was low nutrient content except for phosphorus compounds, both samples had the same soil pH, which was within the range suggested to support the biological degradation process of hydrocarbons, especially by indigenous microorganisms. A. lwoffii and B. subtilis are two species of hydrocarbon degrader bacteria found in both soil samples. B. subtilis and A. lwoffii produce surfactant and emulsion, respectively, as hydrocarbon degraders.^26^,^27^However, due to high concentrations of contaminants in the soil, additional surfactant, nutrient, and aeration (biostimulation) solutions were used in addition to A. lwoffii *and* B. subtilis (bioaugmentation) consortium to support the biodegradation process.

At the beginning of the bioremediation experiment, the number of A. lwoffii and B. subtilis colonies was reduced after the application of a surfactant solution (Tween 80). The total bacterial colonies in the control reactor (1T, 2T) was greater than that in the reactor, which received additional surfactant solutions (1AV and 1TV, 2AV, and 2TV). Surfactants increase the availability of contaminants, especially hydrocarbon compounds, but could worsen the biodegradation process depending on microorganisms.^28^ Application of surfactant solution may cause a decrease in the total number of bacterial colonies, although Tween 80 toxicity only occurs at the beginning of the bioremediation experiment and is at its lowest level after 24 hours.^29^ This was in line with the increase in CO_2_ concentration, decrease in soil pH, rise in total colony number, indicating a significant presence of microorganism activity in the initial 14 days of the experiment. Bacteria use hydrocarbons as a primary energy source to increase their population. Generally, water and CO_2_ are the final products of the biodegradation activity of hydrocarbon compounds.^30^ During the decomposition process, hydrocarbon compounds are broken down to form alcohols and fatty acids for metabolism use. The presence of products, including CO_2_, alcohol, and fatty acids between soil pores, is the leading cause of soil pH decrease during the bioremediation process.

From the beginning of the bioremediation experiment, the concentration of CO_2_ and the number of A. lwoffii *and* B. subtilis colonies decreased, but the soil pH increased to neutral conditions. These conditions indicated a decrease in the activity of hydrocarbon-decomposing bacteria. Total petroleum hydrocarbondecomposing bacteria quickly decompose and consume hydrocarbon compounds during the first three weeks from the beginning of incubation.^31^

A. lwoffii *and* B. subtilis are heterotrophic aerobic bacteria.^32^,^33^ Increasing oxygen between soil pores by intermittent aeration was advantageous and enhanced the performance of the bacteria. According to the study, intermittent aeration had a slightly higher TPH removal, but was not regarded as a significant difference compared to the absence of intermittent aeration, except on 2TV, in which TPH removal was better than 2AV. In addition, the presence of intermittent aeration was unfavorable to improve bioremediation performance in reducing Mn concentrations. The increased presence of oxygen due to intermittent aeration caused the oxidation of Mn^2+^ (bioavailable) to Mn^4+^ (non-bioavailable form).

Additionally, there was high removal of Fe from Soil 1 and Soil 2 samples. However, TPH removal in Soil 1 was higher than in Soil 2. Magnesium, potassium, Fe, and Mn elements are the main factors in surfactant production by B. subtilis.^26^ The conditions in Soil 1 were more favorable for stimulating B. subtilis and A. lwoffii to produce more biosurfactants. This could be a cause for better performance of bioaugmentation in Soil 1 compared to Soil 2.

In contrast to Mg and Fe, which were greater in Soil 1, Soil 2 had higher Mn content *(Table 1).* The amount of surfactant produced by B. subtilis is greater when it grows on media containing Mn compared to B. subtilis on Fe, although the total difference was not significant.^26^

Thus, the results of the present study showed that B. subtilis, A. lwoffii, and indigenous bacteria in Soil 2 samples naturally had better biodegradation performance than Soil 1, as demonstrated by higher TPH removal in 2T (control of Soil 2) than in 1T (control of Soil 1).

## Conclusions

Ship dismantling activities in Tanjungjati Village, Bangkalan district, Indonesia have led to soil contamination by hydrocarbons and metals, especially Fe and Mn. The combination of surfactant solution, bioaugmentation (A. lwoffii and B. subtilis), and biostimulation provided the best results in the removal of hydrocarbons and metals. Using the combination of these treatments, the results for TPH, Fe, and Mn removal obtained in Soil 1 were 69.62%, 87.10%, 29%, and were higher than in Soil 2 samples at 28.80%, 65.10%, 57.38% removal, respectively. Therefore, the concentration of Fe and Mn in Soil 1 and 2 samples may be naturally reduced without the combination of treatments. Higher TPH removal in soils treated with bioaugmentation indicated that B. subtilis and A. lwoffii consortium are potential hydrocarbon degraders.

## References

[1] Fariya S, Manfaat D, Suastika K (2016). Technical analysis of the development of ship recycling yard in Indonesia.

[2] Rabbi HR, Rahman A (2017). Ship breaking and recycling industry of Bangladesh; issues and challenges. Procedia Eng [Internet].

[3] (2003). Technical guidelines for the environmentally sound management of the full and partial dismantling of ships: Basel Convention on the control of transboundary movements of hazardous wastes and their disposal [Internet].

[4] Fahruddin (2010). Bioteknologi lingkungan [Environmental biotechnology].

[5] King RB, Long GM, Sheldon JK (1997). Practical environmental bioremediation: the field guide.

[6] Mirsal I (2008). Soil pollution: origin, monitoring & remediation.

[7] Abdulsalam S, Bugaje IM, Adefila SS, Ibrahim S (2011). Comparison of biostimulation and bioaugmentation for remediation of soil contaminated with spent motor oil. Int J Eviron Sci Technol [Internet].

[8] Adeyemo AJ, Mello JW, Agele SO (2015). Bioremediation of three Brazilian soils contaminated with used lubricating oil. Int J Plant Soil Sci.

[9] Safdari MS, Kariminia HR, Rahmati M, Fazlollahi F, Polasko A, Mahendra S, Wilding WV, Fletcher TH (2018). Development of bioreactors for comparative study of natural attenuation, biostimulation, and bioaugmentation of petroleum-hydrocarbon contaminated soil. J Hazard Mater [Internet].

[10] Pranowo PP, Titah HS (2016). Isolation and screening of diesel-degrading bacteria from the diesel contaminated seawater at Kenjeran Beach, Surabaya. EnvironmentAsia.

[11] Titah HS, Pratikno H, Moesriati A, Putera RI, Imron MF (2017). Identification of diesel resistant bacteria that isolated from ship dismantling area in Madura Coastal.

[12] Sakthipriya N, Doble M, Sangwai JS (2015). Bioremediation of coastal and marine pollution due to crude oil using a microorganism Bacillus subtilis. Procedia Eng [Internet].

[13] Syed S, Chinthala P (2015). Heavy metal detoxification by different bacillus species isolated from solar salterns. Scientifica [Internet].

[14] Costa AC, Duta FP (2001). Bioaccumulation of copper, zinc, cadmium and lead by Bacillus sp., Bacillus cereus, Bacillus sphaericus and Bacillus subtilis. Braz J Microbiol [Internet].

[15] Hamzah A, Rabu A, Azmy RFHR, Yussoff NA (2010). Isolation and characterization of bacteria degrading Sumandak and South Angsi Oils. Sains Malays.

[16] Bahobail A, Gad El-Rab SM, Amin GA (2016). Locally isolated bacterial strains with multiple degradation potential capabilities on petroleum hydrocarbon pollutants. Adv Microbiol.

[17] Sulaeman E, Prasetyo BH, Santoso D, Retno WL (2009). Technical guidelines for chemical analysis of soil, plant, water and fertilizer.

[18] Villalobos M, Avila-Forcada AP, Gutierrez-Ruiz ME (2008). An improved gravimetric method to determine total petroleum hydrocarbons in contaminated soils. Water Air Soil Pollut [Internet].

[19] de Vries W, Lofts S, Tipping E, Meili M, Groenenberg JE, Schutze G, Ware G (2007). Impact of soil properties on critical concentrations of cadmium, lead, copper, zinc, and mercury in soil and soil solution in view of ecotoxicological effects. Reviews of environmental contamination and toxicology.

[20] Yan J, Quan G, Ding C (2013). Effects of the combined pollution of lead and cadmium on soil urease activity and nitrification. Procedia Environ Sci [Internet].

[21] Elgala AM, Ismail AS, Ossman MA (1986). Critical levels of iron, manganese and zinc in Egyptian soils. J Plant Nutr [Internet].

[22] (2003). State Minister of Environment Decree No.128/2003: Guideline for Hydrocarbon Waste and Contaminated Soil Treatment Using Biological Method.

[23] Kostecki PT, Calabrese EJ (1992). Contaminated soils: diesel fuel contamination.

[24] Kayode J, Oyedeji AA, Olowoyo O (2009). Evaluation of the effects of pollution with spent lubricating oil on the physical and chemical properties of soil. Pac J Sci Technol.

[25] Nwite JN, Alu M (2015). Effect of different levels of spent engine oil on soil properties, grain yield of maize and its heavy metal uptake in Abakaliki, Southeastern Nigeria. J Soil Sci Environ Manag.

[26] Wei Y, Lai C, Chang J (2007). Using Taguchi experimental design methods to optimize trace element composition for enhanced surfactin production by Bacillus subtilis ATCC 21332. Process Biochem [Internet].

[27] Nakar D, Gutnick DL (2003). Involvement of a protein tyrosine kinase in production of the polymeric bioemulsifier emulsan from the oil-degrading strain Acinetobacter lwoffii RAG-1. J Bacteriol [Internet].

[28] Cheng M, Zeng G, Huang D, Lai C, Zhang C, Liu Y (2017). Advantages and challenges of Tween 80 surfactantenhanced technologies for the remediation of soils contaminated with hydrophobic organic compounds. Chem Eng J [Internet].

[29] Bautista LF, Sanz R, Molina MC, Gonzalez N, Sanchez D (2009). Effect of different non-ionic surfactants on the biodegradation of PAHs by diverse aerobic bacteria. Int Biodeterior Biodegradation.

[30] Prakash A, Bisht S, Singh J, Teotia P, Kela R, Kumar V (2014). Biodegradation potential of petroleum hydrocarbons by bacteria and mixed bacterial consortium isolated from contaminated sites. Turk J Eng Environ Sci.

[31] Wu M, Li W, Dick WA, Ye X, Chen K, Kost D, Chen L (2017). Bioremediation of hydrocarbon degradation in a petroleum-contaminated soil and microbial population and activity determination. Chemosphere [Internet].

[32] Yang XP, Wang SM, Z ang DW, Zhou LX (2011). Isolation and nitrogen removal characteristics of an aerobic heterotrophic nitrifying-denitrifying bacterium, Bacillus subtilis A1. Bioresour Technol [Internet].

[33] Baumann P (1968). Isolation of Acinetobacter from soil and water. J Bacteriol [Internet].

